# Association of SAP130/SF3b-3 with Cullin-RING ubiquitin ligase complexes and its regulation by the COP9 signalosome

**DOI:** 10.1186/1471-2091-9-1

**Published:** 2008-01-03

**Authors:** Suchithra Menon, Tomohiko Tsuge, Naoshi Dohmae, Koji Takio, Ning Wei

**Affiliations:** 1 Department of Molecular, Cellular and Developmental Biology, Yale University, New Haven, Connecticut, USA; 2 Institute for Chemical Research, Kyoto University, Gokasho, Uji Kyoto 611-0011, Japan; 3 Biomolecular Characterization, RIKEN, 2-1 Hirosawa, Wako Saitama 351-0198, Japan

## Abstract

**Background:**

Cullin-RING ubiquitin E3 ligases (CRLs) are regulated by modification of an ubiquitin-like protein, Nedd8 (also known as Rub1) on the cullin subunit. Neddylation is shown to facilitate E3 complex assembly; while un-neddylated cullins are bound by CAND1 that prevents recruitment of the substrates. The level of Nedd8 modification is critically dependent on the COP9 signalosome (CSN), an eight-subunit protein complex containing Nedd8 isopeptidase activity.

**Results:**

We report isolation of SAP130 (SF3b-3) as a CSN1 interacting protein. SAP130 is homologous to DDB1, and is a component of SF3b RNA splicing complex and STAGA/TFTC transcription complexes, but its specific function within these complexes is unknown. We show that SAP130 can interact with a variety of cullin proteins. It forms tertiary complexes with fully assembled CRL E3 complexes such as SCF^Skp2^, Elongin B/C -Cul2- VHL and Cul4-DDB complex by binding to both N-terminal and C-terminal domain of cullins. SAP130 preferentially associates with neddylated cullins *in vivo*. However knock-down of CAND1 abolished this preference and increased association of SAP130 with Cul2. Furthermore, we provide evidence that CSN regulates SAP130-Cul2 interaction and SAP130-associated polyubiquitinating activity.

**Conclusion:**

SAP130 is a cullin binding protein that is likely involved in the Nedd8 pathway. The association of SAP130 with various cullin member proteins such as Cul1, Cul2 and Cul4A is modulated by CAND1 and CSN. As an established component of transcription and RNA processing complexes, we hypothesis that SAP130 may link CRL mediated ubiquitination to gene expression.

## Background

Cullin-RING ubiquitin ligase (CRL) family of E3 enzymes participates in diverse cellular and physiological processes [1]. Each CRL complex contains a cullin family member that serves as a scaffold to assemble a functional E3 complex. The C-terminal globular domain of cullin interacts with the small RING protein Rbx1 (Roc1 or Hrt1) forming the catalytic core, while the substrate-recognizing module assembles at the N-terminal cullin repeats domain [2,3]. In SCF (Skp1-Cullin1-F-box protein) complexes, the substrate-recognizing module consisting of Skp1 and an F-box protein such as Skp2 interacts specifically with Cul1 [4]. Similarly, Elongin B/C-VHL complex interacts with Cul2 [5,6], the BTB domain substrate adaptor binds to Cul3, while DDB1 serves as a Cul4 adaptor [7-9]. DDB1 belongs to a family of proteins with significant sequence homology [10] that includes SAP130/SF3b-3, a component of transcription and RNA splicing complex [11] and CPSF160, cleavage and polyadenylation specificity factor [12]. The latter two proteins have not been reported to have functions involving the ubiquitin system.

Human cells express seven different cullins, most of which if not all can be modified by an ubiquitin-like protein Nedd8 (or Rub1) on a conserved lysine residue near the C-terminus of the cullin subunit [13]. Nedd8 conjugation to cullins (neddylation) is catalyzed by Nedd8-specific E1 and E2 enzymes [14], while its removal (de-neddylation) is mediated by COP9 signalosome (CSN) [15,16]. The de-neddylated (or un-neddylated) cullins are specifically bound by CAND1 [17-19]. Neddylation is shown to facilitate polyubiquitination by CRLs, while de-neddylation is necessary to maintain the stability of CRL components [13,20]. It was recently shown that Skp1-Skp2 complex triggers dissociation of CAND1 from Cul1 and, when charged with substrate p27, induces Cul1 neddylation by inhibiting CSN mediated de-neddylation [21]. This study shows that the substrate-binding module with the charged substrate can drive cullin neddylation.

CSN is a functionally pleiotropic complex composed of eight subunits designated CSN1 to CSN8, each having unique roles and specific functions [22]. In addition to the de-neddylase activity that involves CSN2 and CSN5/JAB1 [23,24], CSN also interacts and recruits de-ubiquitin enzymes and protein kinases [25,26]. Deletions of CSN cause drastic alterations in the gene expression profile and early lethality of multi-cellular model organisms such as *Arabidopsis*, Drosophila, and mouse [22].

We have shown previously that CSN1 central and C-terminal regions are necessary for CSN complex integrity in both plant and animal cells [27,28]. The N-terminal domain (NTD) of CSN1 is not involved in complex assembly but carries an activity that inhibits AP-1 dependent transcription in mammalian cells [27]. In *Arabidopsis*, deletion of CSN1-NTD causes early lethality despite that the mutant (*fus6/C231*) can assemble a CSN complex (CSN^S1-C231^) [28]. To understand the functions associated with CSN1-NTD, we initiated a search for proteins interacting with this domain. We report here identification of SAP130/SF3b3, a member of DDB1 family proteins and an established component of transcription complex and RNA processing complex. Our data show that SAP130 is a general cullin binding protein that normally associates with neddylated form of endogenous cullins *in vivo*. The preference of SAP130 for neddylated cullins is dependent on CAND1, a protein that specifically binds un-neddylated cullins. SAP130 predominantly binds C-terminal domain of cullin and forms tertiary complex with fully assembled CRLs.

## Results

### SAP130/SF3b3 is a CSN1-NTD binding protein

**Figure 1:**
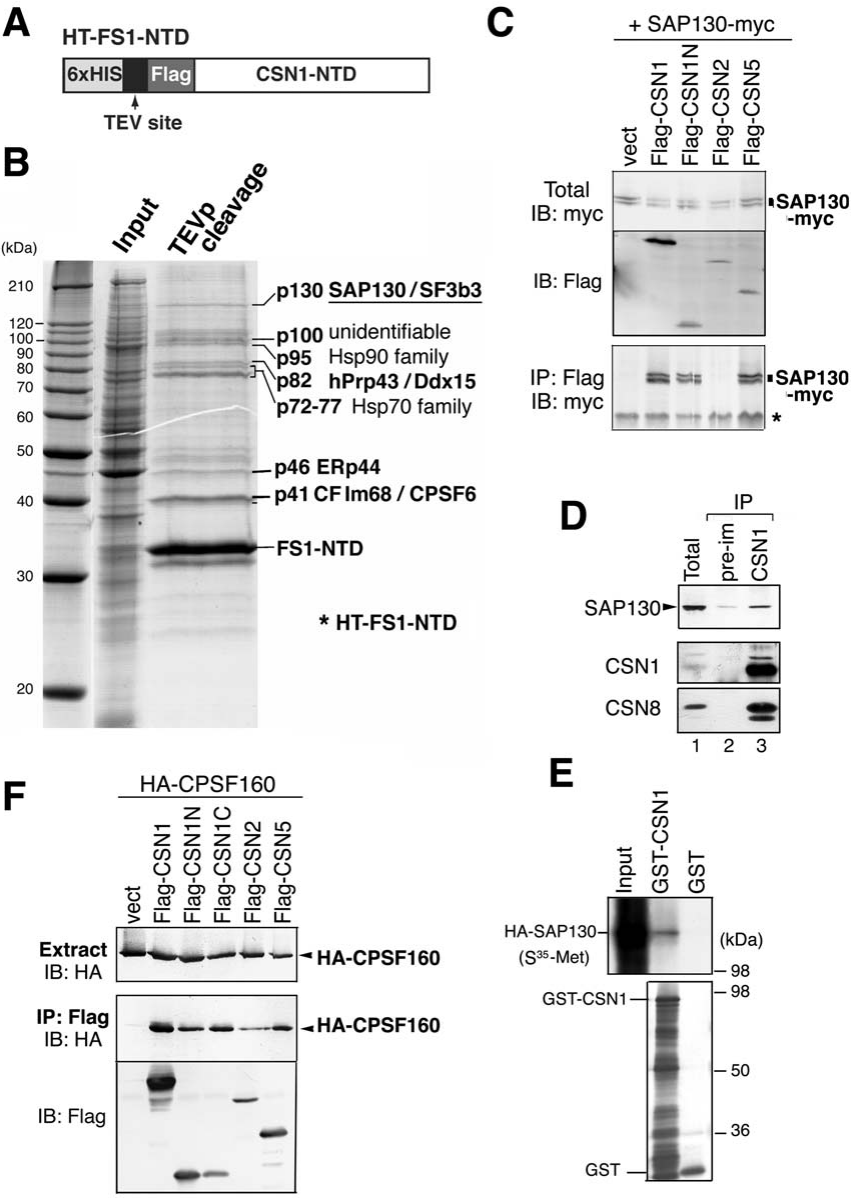
**SAP130 is a CSN1 interacting protein**. **(A) **Diagram of bait HT-FS1-NTD. From the N-terminus, this protein contains a His-tag (6 × HIS), Tobacco Etch Virus protease recognition and cleavage site (TEV site) and Flag tagged CSN1-NTD (1–196 aa). (**B) **Coomassie Blue staining gel showing the input NIH3T3 cell lysate and proteins eluted after TEV protease cleavage. Identity of corresponding proteins is labeled. **(C) **SAP130-myc was co-transfected in HeLa cells with empty vector (lane 1) or Flag-tagged CSN subunits as indicated. Total cell extract and the Flag immunoprecipitated (IP) proteins were probed with anti-myc antibody. **(D) **CSN1 was immunoprecipitated from DRB treated NIH3T3 nuclear extract. Endogenous SAP130 was enriched in CSN1 IP compared to pre-immune serum (pre-im). **(E) **HA-SAP130 was *in vitro *translated and S^35^-Met labeled in rabbit reticulocyte lysate. The samples were incubated with recombinant GST or GST-CSN1 proteins (1 μg each) for 30 min. Following GST pull-down, samples were analyzed by autoradiogram (upper panel) or anti-GST immunoblotting (lower panel). **(F) **HA-CPSF160 was transiently co-transfected in HeLa cells with empty vector (lane 1) or Flag-tagged CSN subunits. Total cell extract and the Flag immunoprecipitated proteins were probed with anti-HA antibody.

To isolate CSN1-NTD interacting proteins, we took a biochemical approach previously described by Lyapina *et al. *[15]. The bait protein HT-FS1-NTD, which contains an N-terminus 6 × His-tag linked to the TEV protease cleavage site upstream of a Flag-tagged CSN1-NTD (1–200 aa) (Fig. 1A), was immobilized and incubated with NIH3T3 whole cell extract. Upon TEV protease cleavage, CSN1-NTD and its bound proteins were gently eluted and visualized by coomassie blue staining (Fig. 1B). Individual protein bands were excised and identified by mass-spectrometry and peptide sequencing (Fig. 1B and Table 1). As anticipated, all of these proteins were substoichiometric to the bait, suggesting that the interaction with CSN1-NTD is weak or transient in contrast to stable subunit-subunit interactions within CSN complex.

**Table 1:** Identification of CSN1-NTD interacting proteins

Band No.	Peptides	Protein identity (accession No.)	Putative function
p130	(K)TPVEEVPAAIAPFQGRVLIGVGK (K)QQEIVVSRGK	SAP130/SF3b-3, (BC011412)	RNA splicing, transcription
p100	(K)GFRQGPQPFTQQK (K)EATQTVAADTRP	Un-assignable	
p95	(K)YYEQFSK	HSP90, silkworm (BAB41209)	Chaperon
p82	(K)REVDDLGPEVGDIK (K)VYNPRIRVESLLVTAISK	Ddx15/mDEAH9/prp43 (O35286)	RNA splicing
p72-77	(K)SQIHDIVLVGGSTRIPK (K)HWPFMVVNDAGRPK (K)LLQDFFNGK (K)TVQNAVITVPAYFNDSQRQATK	Hsc70 family, Hspa8 (NP_112442)	Chaperon
p46	(K)LHREFHYGPDPQPVMQLDG (K)EGAVATRFHITKYPTLK	CG9911 Drosophila (AAF48579) similar to mouse ERp44	oxidative protein folding in the ER
p41	(K)FFENRANGQSK (K)EIHGQHPVVTLPTK (K)RALNQFESQ	Insect homolog (XP_313937) of CPSF6/CFIm68	mRNA 3' processing

Besides heat shock 70 and 90 families of proteins, the following proteins were identified: SAP130/SF3b-3, Ddx15, CF Im68 and ERp44 (Fig. 1B). SAP130 is a component of SF3b complex, which is an integral part of 17S U2 snRNP particle [11,29]. SAP130, but not other SF3b subunits, is also a stoichiometric component of GCN5-containing transcription complexes such as STAGA and TFTC complexes [TATA binding protein (TBP)-free TAF_II _complexes] [30,31]. Ddx15 (hPrp43 or mDEAH9) and CF Im68 are involved in RNA processing, while ERp44 plays a key role in thiol-mediated retention in ER.

We are particularly interested in SAP130 because it is highly homologous to DDB1 [10], a known component of Cul4-based ubiquitin ligase complex that is tightly regulated by the CSN complex [32,33]. To verify interaction of SAP130 with CSN1, SAP130-myc was transiently expressed with representative CSN subunits in HeLa cells (Fig. 1C). Flag-CSN1, CSN1N (1–196 aa) and Flag-CSN5, but not Flag-CSN2, readily co-precipitated SAP130-myc. Immunoprecipitation of endogenous CSN1 from nuclear extract weakly but consistently pulled down endogenous SAP130 (Fig. 1D). In addition, recombinant GST-CSN1, but not GST itself, could pull down *in vitro *translated SAP130 from the lysate (Fig. 1E). Similarly, another member of DDB1 family, CPSF160 can also co-immunoprecipitate CSN subunits when transiently expressed in cultured cells (Fig. 1F). Therefore, all three members of the DDB1 family in humans can interact with CSN.

### Preferential association of SAP130 with neddylated cullins is dependent on CAND1

**Figure 2:**
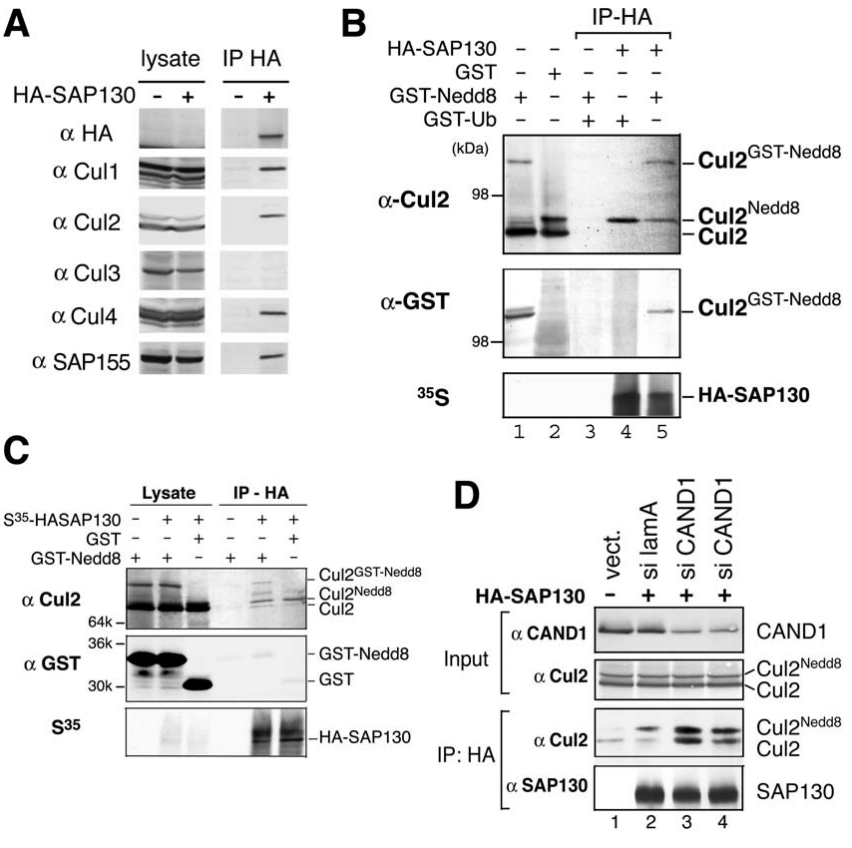
**SAP130 interacts with Nedd8 modified cullins**. (**A**) Whole cell extracts (lysate) and anti-HA immunoprecipitate (IP HA) from HEK293 cells transiently transfected with empty vector (-) or HA-SAP130 (+) were analyzed by immunoblotting using the antibodies against cullins or SAP155, a component of SF3b RNA-splicing complex. **(B) **Rabbit reticulocyte lysate (RRL) containing *in vitro *translated HA-SAP130 were incubated with recombinant GST-Nedd8, GST-Ub or GST (1 μg each). HA IP samples were analyzed by anti-Cul2 and anti-GST blots and autoradiogram (S^35^). (**C**) HA-SAP130 was *in vitro *translated in RRL supplemented with GST or GST-Nedd8 (1 μg each). Anti-GST blot shows that free GST-Nedd8, like GST alone, was not associated with HA-SAP130. (**D**) HeLa Cells were transfected with siRNA reagent against CAND1 or laminin-A control. Input lysate or HA IP samples were blotted with anti-CAND1 or Cul2 antibodies. Note that there was weak non-specific binding of un-neddylated Cul2 to HA beads (lane 1).

Given that DDB1 functions as a component of Cul4A ubiquitin ligase complex, we investigated possible interaction of SAP130 with cullins. Transiently expressed SAP130 co-immunoprecipitated endogenous Cul1, Cul2, Cul4 and SAP155, a known component of SF3b complex (Fig. 2A). We noticed that SAP130 appeared to pull-down only the slower migrating cullin band that normally corresponds to neddylated form of cullins. To definitively verify this, SAP130 was *in vitro *translated in rabbit reticulocyte lysate (RRL) supplemented with recombinant GST-Nedd8, GST-ubiquitin or GST proteins. In this system, GST-Nedd8 can be conjugated to endogenous Cul2 in the lysate generating Cul2^GST-Nedd8 ^(Fig. 2B, lanes 1 and 5). Both anti-Cul2 and anti-GST blots showed that SAP130 specifically co-immunoprecipitated Cul2^GST-Nedd8 ^and Cul2^Nedd8^, but not un-neddylated Cul2 (Fig. 2B, lanes 4 and 5). This result explicitly demonstrates that SAP130 preferentially associates with neddylated Cul2 in the lysate. Taken together the data in Figures 2A and 2B, SAP130 most likely interacts with other neddylated cullins such as Cul1 and Cul4. The observation that Cul3 was not co-immunoprecipitated by SAP130 as shown in Figure 2A was probably due to the fact that neddylated Cul3 could not be detected in the total cell extract by the anti-Cul3 antibody (Fig. 2A). It should be mentioned that because neddylated cullins tend to be unstable, the experiments shown above were carried out in the presence of proteasome inhibitor MG132 (see Methods).

We next asked whether SAP130 binds Nedd8 itself by examining un-conjugated GST-Nedd8 monomer in SAP130 immunoprecipitates. As shown in Figure 2C, GST-Nedd8, similar to GST itself, was not bound by SAP130, indicating that SAP130 did not have specific affinity to un-conjugated Nedd8.

It has been shown that CAND1 (also known as TIP120A) specifically binds un-neddylated cullins forming stable CAND1-cullin complexes in the cells [17-19]. To determine whether the level of CAND1 may affect the selective association of SAP130 with neddylated cullins, we knocked down CAND1 level in HeLa cells by siRNA. Reduction of CAND1 amount did not affect the steady state neddylation level of Cul2, however SAP130 no longer exhibited neddylation preference as it co-immunoprecipitated both neddylated and un-neddylated Cul2 (Fig. 2D). Concomitant with the loss of neddylation preference was an increase in the amount of Cul2 that was associated with SAP130 (Lanes 3 and 4). This data suggests that CAND1 inhibited SAP130-Cul2 interaction and that CAND1 was necessary for preferential association of SAP130 with neddylated Cul2 in the cells. Although the precise mechanism is not understood, it seems possible that CAND1 sequesters un-neddylated cullins from SAP130. Regardless, this data showed that neddylation is not necessary for SAP130 to bind cullins and that SAP130 has no intrinsic specificity for Nedd8.

### SAP130 forms tertiary complexes with CRLs and associates with ubiquitin E3 activity

**Figure 3:**
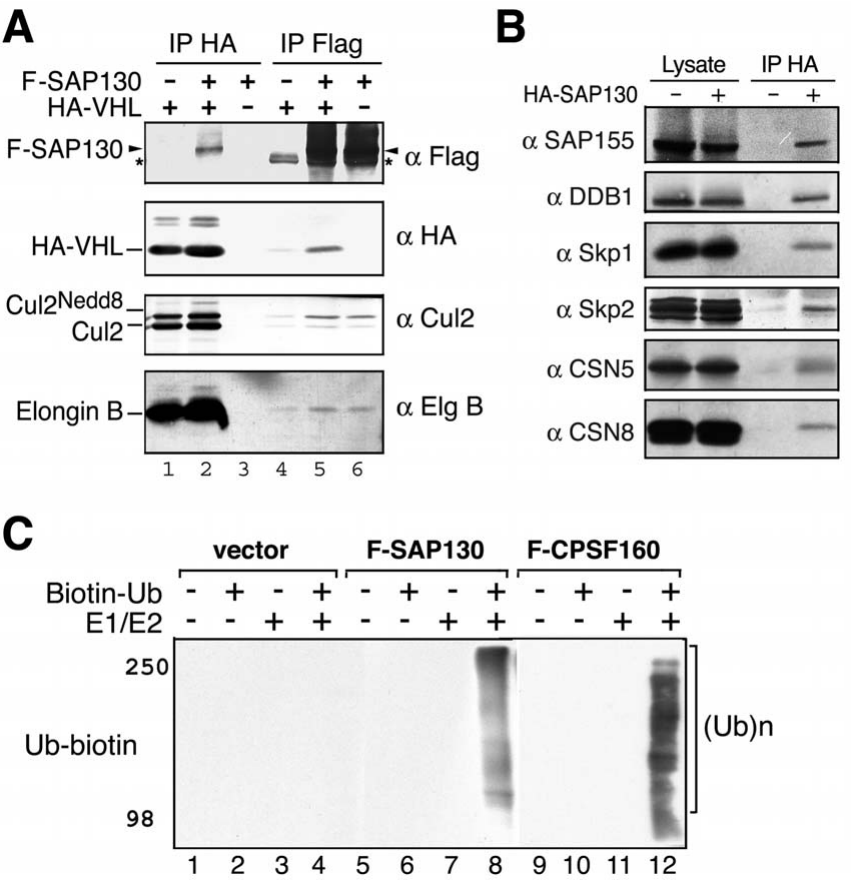
**SAP130 forms tertiary complexes with CRL substrate adaptors and is associated with E3 activity**. **(A) **HEK293 cells were transfected with HA-VHL or Flag-SAP130. Anti-HA and anti-Flag IP samples were blotted with indicated antibodies. An asterisk (*) indicates a non-specific band in Flag IP. (**B**) HA-SAP130 was expressed in HEK293 cells. Anti-HA IP samples were probed with indicated antibodies. **(C) **Flag immunocomplex isolated from HEK293 cells expressing empty vector, Flag-SAP130 or Flag-CPSF160 were incubated with N-terminal biotin-labeled ubiquitin, E1 and E2 (UbcH5b) as indicated. Polyubiquitination was determined by the detection of biotin-Ub.

Next we asked whether SAP130 could form tertiary complex with the substrate-binding components of CRLs. The Cul2 substrate receptor HA-VHL and Flag-SAP130 were expressed in HEK293 cells. VHL and SAP130 clearly co-immunoprecipitated each other (Fig. 3A, lanes 2 and 5) supporting the idea that SAP130 can form a tertiary complex with the Cul2-VHL E3 complex. Noticeably, while VHL pulled down both neddylated and un-neddylated Cul2, SAP130 pulled down predominantly the neddylated Cul2. In addition, SAP130 also co-precipitated endogenous Skp1 and Skp2, as well as Cul4 adaptor DDB1 (Fig. 3B) and a transiently expressed DDB2 (data not shown). These results suggest that in contrast to CAND1, SAP130 is able to form tertiary complexes with different CRL E3s including SCF, Cul2-VHL and Cul4-DDB1 complexes.

We reasoned that by binding to active CRLs, SAP130 may associate with E3 ubiquitin ligase activities. Flag-SAP130 and Flag-CPSF160 immunocomplexes were isolated from cultured cells and *in vitro *E3 activity assay was performed (Fig. 3C). In this assay, the anti-ubiquitin blot was used to detect the polyubiquitin products generated by the immunocomplexes. As anticipated, both SAP130 and CPSF160 immunocomplexes exhibited robust ubiquitin E1/E2 dependent polyubiquitinating activities *in vitro *(Fig. 3C).

### SAP130 binding domains in cullins

**Figure 4:**
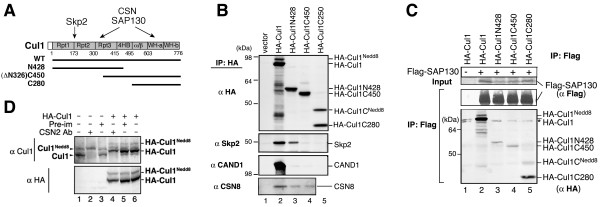
**Interaction of SAP130 with HA-Cul1**. **(A) **A diagram indicating the Cul1 truncation constructs and the interaction results. **(B) **HA-Cul1 and its truncations HA-Cul1N428, HA-Cul1C450 and HA-Cul1C280 were transiently expressed in HEK293 cells. The HA-IP samples were blotted with indicated antibodies. **(C) **HA-Cul1 and its truncations as indicated were transfected into Flag-SAP130 stable cells. Flag IP was performed and samples were probed with anti-HA antibody. **(D) **HeLa cells were transfected with HA-Cul1 or vector. Cell extracts were incubated with anti-CSN2 antibody or pre-immune serum for 15 min at room temperature and then blotted for Cul1 or HA. Arrowheads indicate endogenous Cul1 and the lines on the right indicate overexpressed HA-Cul1.

In CRL complexes, the substrate-binding module assembles at the N-terminal cullin repeats, while CAND1 binds to both N- and C-terminal domains of cullins [2,34]. To define the SAP130 binding domains on cullins, we constructed the following truncations in HA-Cul1 (Fig. 4A): Cul1N428 (1–428 aa), Cul1C450 (326–776 aa) and Cul1C280 (496–776 aa). As expected, Skp2 was co-precipitated by full-length Cul1 and Cul1N428 but not Cul1C450 or Cul1C280 (Fig. 4B). CAND1 only interacted with full-length Cul1 but none of the truncations, while CSN8 could associate with both the N-terminal and C-terminal Cul1 deletions (Fig. 4B). SAP130 strongly interacted with Cul1 C-terminal fragment C280 and was also associated with C450 and N428 (Fig. 4C). This result shows that SAP130 can bind to both N-terminal as well as the C-terminal domains of Cul1.

We noticed that while SAP130 preferred neddylated form of endogenous Cul1, it could bind both neddylated and un-neddylated forms of ectopically expressed HA-Cul1 (Fig. 4C lane 2). To understand how endogenous Cul1 may differ from ectopically expressed HA-Cul1 in neddylation dynamics, we compared the changes in the neddylation level in response to CSN inactivation (Fig. 4D). We previously reported that the de-neddylation activity of CSN could be blocked by anti-CSN2 antibody [24,35]. Specifically, incubation of the cell extract with anti-CSN2 antibody blocks the de-neddylation reaction but not the cellular neddylation activity, causing neddylated cullins to accumulate. HeLa cell extracts containing over-expressed HA-Cul1 or vector were incubated with anti-CSN2 antibody or a pre-immune serum. While endogenous Cul1 became completely neddylated by anti-CSN2 treatment (Fig 4D, lanes 2 and 4), the neddylation level of HA-Cul1 did not change (lane 4). This result suggests that the neddylation reaction on HA-Cul1 was slower or deficient compared to endogenous Cul1. Under the identical condition when de-neddylation was blocked, HA-Cul1 failed to be fully neddylated like endogenous Cul1 (Fig. 4D, lane 4), suggesting that overexpressed HA-Cul1 is a poor neddylation substrates *in vivo*. We reasoned that HA-Cul1 overexpression system is unsuitable to study the neddylation and de-neddylation dynamics. However, this system is still valid for defining the Cul1 domains involved in SAP130 binding. In fact, SAP130 interaction with cullins does not require neddylation as shown earlier.

**Figure 5:**
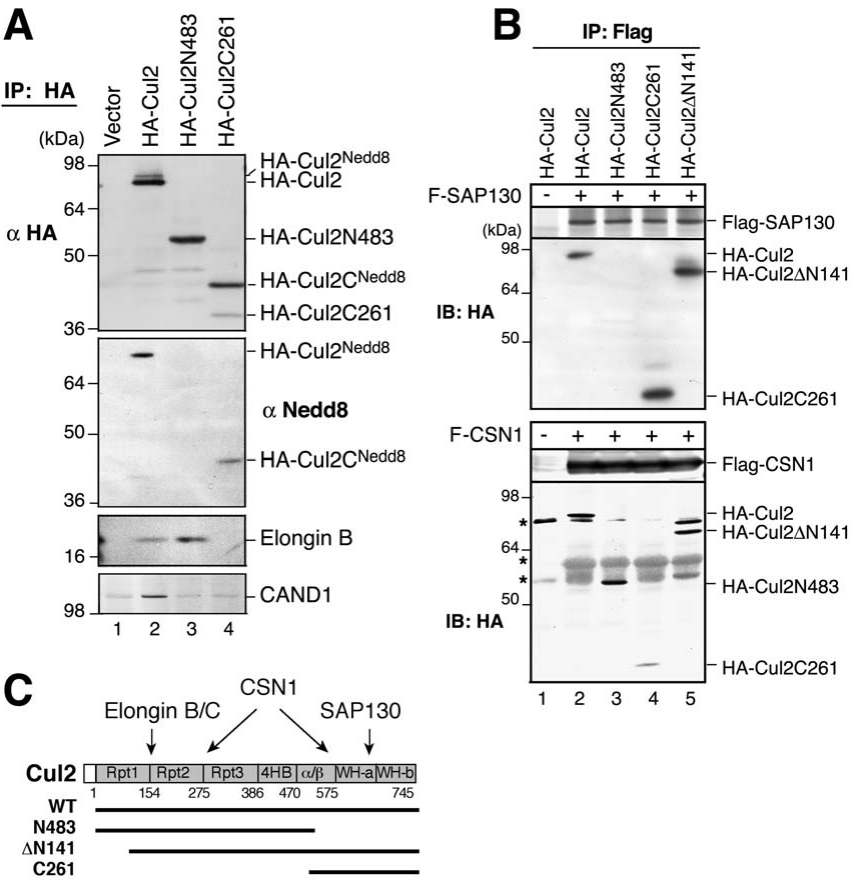
**SAP130 binds to Cul2 CTD**. **(A) **HA-Cul2 and its truncations HA-Cul2N483 and HA-Cul2C261 were transiently transfected in HEK293 cells. The HA-IP samples were blotted with antibodies against HA, Nedd8, Elongin B or CAND1. Nedd8 modified and un-modified Cul2 proteins are indicated. **(B) **HA-Cul2 and its truncations as indicated were transfected into Flag-SAP130 stable cells (upper panel) or HEK293 cells along with Flag-CSN1 (bottom panel). Flag IP was performed and samples were blotted using anti-HA antibody. **(C) **A diagram indicating the Cul2 truncation constructs and the interaction results from **(A) **and **(B)**.

To extend the observations on SAP130-Cul1 interaction, we carried out a similar experiment on Cul2 truncations (Fig. 5). HA-Cul2C261 (484–745 aa) containing the WH-a and WH-b domains and the Nedd8 attachment site (K689) can be effectively neddylated, as indicated by the Nedd8 blot after HA-precipitation (Fig. 5A, lane 4). As predicted, both the full-length Cul2 and the N483 fragment could pull-down Elongin B, while C261 could not (Fig. 5A). This result is consistent with our understanding that the N-terminal region of Cul2 is necessary and sufficient for binding to Elongin B/C-VHL substrate-binding module. As in Cul1 binding experiment, CAND1 was associated only with full-length Cul2 but not with any of the terminal deletions (Fig. 5A, bottom panel).

We found that SAP130 specifically pulled down the C-terminal fragments C261 and ΔN141 (142–745 aa), but not N483 (Fig. 5B, upper panel), indicating that SAP130 interacted with Cul2 C-terminal WH-a/b domain. In a similar experiment, CSN1 was found to bind HA-Cul2 and all of the truncation mutants tested (Fig. 5B, bottom panel) suggesting that CSN1 or the CSN complex has more than one contact site in Cul2 as in Cul1. These results show that SAP130 predominantly binds the C-terminal domain (CTD) of cullins, but it can also weakly interact with the N-terminal domain; while CSN1 binds to both N- and C-terminal regions of cullins.

### CSN regulates SAP130-cullin interaction

**Figure 6:**
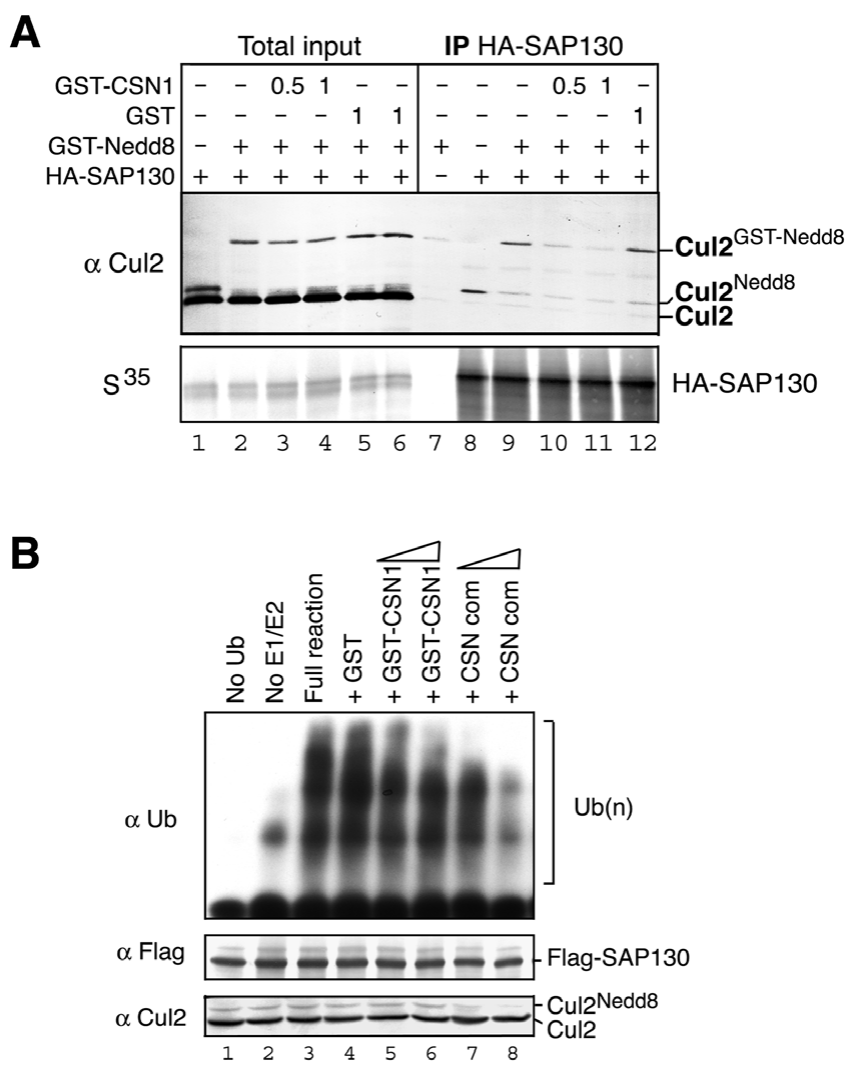
**CSN1 inhibits SAP130-Cul2 interaction and SAP130 associated E3 activity**. **(A) ***In vitro *translated HA-SAP130 in RRL was incubated with GST-Nedd8 (1 μg) and GST-CSN1 (0.5 μg or 1.0 μg) or GST (1.0 μg). The RRL input and HA-IP were analyzed by anti-Cul2 blot. **(B) **Whole cell extract from Flag-SAP130 stable cells were either left untreated or pretreated with GST (2 μg), GST-CSN1 (1 μg and 2 μg) or purified CSN complex from porcine spleen (CSN com). Flag immunocomplex was subsequently isolated and used for *in vitro *E3 activity assay. Polyubiquitin chain was detected by anti-ubiquitin blot. Equal amount of Flag-SAP130 immunocomplex was confirmed by anti-Flag blot. Cul2 blot of the pretreated extract showed reduced neddylation after incubation with CSN complex but not GST-CSN1.

To investigate the role of CSN1 in SAP130-Cul2 interaction, recombinant GST-CSN1 or GST protein was incubated with *in vitro *translated HA-SAP130 in RRL. We found that addition of the recombinant proteins did not affect Cul2 stability or its neddylation level (Fig. 6A). This is consistent with the genetic analysis in *Arabidopsis *that CSN1-NTD is not required for CSN mediated de-neddylation of Cul1 [28]. Notably, the amounts of Cul2^GST-Nedd8 ^that co-precipitated with SAP130 clearly declined when the lysate was supplemented with increasing amounts of GST-CSN1, but not GST (Fig. 6A). This result indicated that excess CSN1 protein could interfere with the interaction between SAP130 and Cul2.

We next determined the effect of CSN1 on SAP130 associated E3 activity. Whole cell extract from a stable Flag-SAP130 line was pre-incubated with recombinant GST-CSN1, GST, or biochemically purified CSN complex. Flag-SAP130 immunocomplex was subsequently isolated and used for the *in vitro *E3 activity assay (Fig. 6B). SAP130 associated polyubiquitination activity was detectably reduced upon addition of excess GST-CSN1 (Fig. 6B, lanes 5, 6) and was dramatically reduced upon addition of CSN complex (lanes 7, 8). The observation that SAP130 associated E3 activity was sensitivity to CSN complex further reinforced the notion that the polyubiquitin chains produced by the SAP130 immunocomplex were mediated by CRLs rather than non-specifically bound E3s. We also found that Cul2 neddylation was reduced upon addition of CSN deneddylase as expected (Bottom panel, lanes 7, 8), but remained unchanged when supplemented with GST-CSN1 (Fig. 6B, bottom panel). Since CSN1 can inhibit SAP130-Cul2 interaction (Fig. 6A), we suggest that CSN1 affects SAP130 associated E3 activity by preventing SAP130-CRL interaction. Taken together, our data indicate that CSN complex negatively regulates SAP130 associated polyubiquitination activity by both cullin de-neddylation and blocking the recruitment of CRL E3 complexes.

## Discussion

CRL ubiquitin E3 ligases are essential ubiquitination machineries in eukaryotes that are involved in a wide array of fundamental processes from genome stability to cell cycle control [1]. Nedd8 attachment and removal from the cullin subunit represents an important mechanism by which CRL functions are regulated [13]. While the enzymes mediating cullin neddylation and de-neddylation have been identified, the regulatory mechanism and the functional consequences of this modification remain obscure. To this end, we have identified SAP130 as a new type of cullin binding protein that interacts with multiple cullins predominantly *via *cullin C-terminal domain. Under normal conditions when CAND1 was present at physiological levels, SAP130 preferred neddylated cullins and associated with fully assembled CRL E3 ligases. SAP130 immunocomplex exhibited a polyubiquitination activity that could be inhibited by excess amount of CSN complex or CSN1 protein.

The role of SAP130 in cullin neddylation pathway remains elusive. Ectopic expression or moderate knock-down of SAP130 in cultured cells did not cause obvious changes in cullin neddylation level (data not shown). We found that neddylation is not an essential signal for SAP130-cullin association, as SAP130 can bind unneddylated cullins when Cul1 or Cul2 were overexpressed in cultured cells or when CAND1 level was knocked down. This result suggests that CAND1 is the key factor that limits SAP130-cullin interaction and renders preferential association of SAP130 with neddylated cullins *in vivo*. Along this line, our data showed that CSN de-neddylase negatively regulates SAP130 associated ubiquitination activity (Fig. 6).

SAP130 does not appear to discriminate between different cullins. This cullin binding capability distinguishes it from conventional CRL adaptors such as Skp1, Elongin B/C and BTB proteins, which exhibit specificity to different cullins by binding to the N-terminal cullin repeats. Nonetheless, since SAP130 weakly associates with the N-terminal region of Cul1 (Fig. 4C), it may act as a substrate adaptor like DDB1 under certain circumstances. It is also possible that SAP130 acts as a general CRL adaptor to assist substrate ubiquitination by binding to cullin C-terminal domain. Along these lines, it is interesting to note that COMMD1, a NF-κB inhibitor, was found to promote ubiquitination of NF-κB by binding to the C-terminal domain of Cul2 in the Elongin B/C-Cul2-SOCS1 E3 complex [36]. COMMD1 regulates the stability of RelA-chromatin complex and the recruitment of Rel-A to the E3 complex in response to stimuli [36,37].

Bornstein *et al. *[21] showed that increased availability of the Cul1 substrate binding components Skp1-Skp2 along with its substrate could trigger Cul1 neddylation. This process is accomplished by a sequential step that involves the dissociation of CAND1 from Cul1 and inactivation of CSN mediated de-neddylation. This finding together with our studies raises the question of whether SAP130 may play an active role in facilitating substrate dependent cullins neddylation, particularly during the gene expression processes.

SAP130 is a known component of RNA processing and transcription complexes. The SF3b complex, which contains SAP130 or SF3b-3, is involved in binding to the intron's branch point of the pre-mRNA during the early step of pre-spliceosome assembly. However, SAP130 is the only subunit of SF3b that does not cross-link to RNA [38]. Similarly, though a component of STAGA/TFTC complexes which bind damaged DNA template and contain histone acetyltransferase activity (HAT) [30,31], SAP130 is neither required for the DNA binding nor the HAT activity [30,39]. To date, the specific role of SAP130 in these complexes is yet unknown.

Increasing evidence show that transcription and co-transcriptional RNA processing are dependent on the ubiquitin-proteasome system and the neddylation pathway. In our recent study on *Csn8 *knockout mice, we found that deletion of Csn8 abolished CSN-mediated deneddylation of cullins such as Cul1, Cul2, and Cul4A [40]. However the upset in Cul1 neddylation level did not compromise degradation of SCF substrates, but caused dramatic transcription defects in a subset of genes [40]. Many CRL components such as F-box proteins, Elongin B/C and VHL have also been implicated in transcription control [41,42]. Our finding that SAP130 associates with cullins raises a tantalizing hypothesis that SAP130 links CRL ubiquitination complexes and the gene expression machinery in a process regulated by CSN. It remains to be elucidated in the future whether SAP130 plays a role in CRL mediated substrate ubiquitination, particularly at the vicinity of RNA processing and transcription complexes.

## Conclusion

Cullin-RING ubiquitin E3 ligases are critical ubiquitination machineries in eukaryotes. In this study, we identified SAP130 as a new cullin binding protein that is likely involved in the Nedd8 pathway. SAP130 interacts with neddylated cullins in vivo in a manner dependent on CAND1 level. SAP130 forms tertiary complexes with active cullin-RING E3 ligases and is associated with polyubiquitination activity that could be modulated by CSN.

## Methods

### Cell lines, growth conditions, transfection and siRNA

NIH3T3, HeLa and HEK 293 cells were maintained in Dulbecco's modified Eagle's (D-MEM) medium with high glucose and supplemented with 10% (v/v) heat inactivated fetal bovine serum in a 37°C humidified incubator containing 5% CO_2_. Flag-SAP130 stable cell line was derived from HEK 293 cells as described [43]. siRNA oligos against CAND1 (Dharmacon) were designed according to Zheng *et al.*[17]. Cells were collected 48 hours post transfection for siRNA experiment and 24 hours for regular plasmid transfection.

### Cell lines, growth conditions, transfection and siRNA

Isolation and identification of CSN1-NTD binding proteins – The fusion protein HT-FS1-NTD was expressed from pFastBacHT vector in a Bac-to Bac^® ^baculorvirus expression system (Invitrogen) using Spodoptera frugiperda (Sf9) insect cells (ATCC). To purify HT-FS1-NTD, 2 ml of infected cell-pellet was sonicated in 6 ml of lysis buffer containing 20 mM Hepes (pH 7.4), 1.5 mM MgCl_2_, 5 mM KCl, 0.5% NP-40, 1 mM DTT, 1 mM PMSF, 1× protease inhibitor cocktail (Roche). The lysate supernatant was incubated with 400 μl of Ni-NTA slurry (Qiagen) in 30 ml binding buffer containing 25 mM Tris-HCl (pH 7.4), 136 mM NaCl, 5 mM KCl, 20 mM immidazole, 1 mM DTT, 1 mM PMSF and 1× protease inhibitor cocktail (Roche) for 3 hr at 4°C. The Ni-NTA beads were washed three times with binding buffer containing 0.1% Tween-20. NIH3T3 cell extract was prepared from a starting material of 2 ml of cell-pellet and sonicated in the lysis buffer. The supernatant was incubated with the HT-FS1-NTD immobilized Ni-NTA beads for 3 hr at 4°C in 15 ml of binding buffer. The beads were then washed and resuspended in 1 ml of rTEV buffer (50 mM Tris-HCl pH 8.0, 0.5 mM EDTA) containing 50 μl TEV protease for 2 hr at 25°C. Samples were separated by SDS-PAGE and stained by coomassie blue and characterized by western blots. To identify the proteins, coomassie-stained bands were excised and treated with 0.2 μg of Achromobacter protease I (API; a gift from Dr. Masaki, Ibaraki University). The peptides were analyzed by mass spectrometry and peptide micro-sequencing. Experimental details on the procedures are available upon request.

### Plasmid constructs and recombinant proteins

The pHT-FS1-NTD plasmid was constructed by inserting the NcoI – SmaI fragment from Flag-CSN1 [27] into the pFastBacHT vector at NcoI – XbaI (blunt-ended) site. Construction strategy for HA-Cul1 and HA-Cul2 deletion clones are available upon request. For pGST-CSN1, the BamHI (blunt-ended)-XhoI fragment from pcDNA3-CSN1 [27] was inserted into pGEX-4T-1 vector at NotI (blund-ended)-XhoI sites.

GST-CSN1, GST-Nedd8, GST-Ub and GST proteins were expressed in E. coli, purified on glutathione sepharose beads (Amersham Bioscience) and eluted with 10 mM reduced glutathione. The samples were dialyzed against a buffer containing 20 mM Tris-HCl pH 7.5 and 20 mM NaCl.

### Immunoprecipitation and antibodies

Transfections were performed using Lipofectamine™2000 (Invitrogen). For immunoprecipitation, 10 μM MG132 (Sigma) was added for 3–5 hrs before collecting the samples. Whole cell extract was prepared in lysis buffer [50 mM Tris-HCl pH 7.5, 150 mM NaCl, 5 mM MgCl_2_, 0.4% NP-40, 1 mM DTT, 1 mM PMSF, 10 μM MG132, 1× protease inhibitor cocktail (Roche)] by sonication.

Antibodies against SAP130 and SAP155 [11] were kindly provided by Dr. Robin Reed (Harvard School of Medicine). Antibodies against CAND1 and Skp2 were kindly provided by Dr. Hui Zhang (Yale School of Medicine) and anti-Cul3 was provided by Dr. Yue Xiong (University of North Carolina). Anti-CSN1, CSN2 and CSN8 have been described [24]. The rabbit antibody anti-Cul4A was raised against the C-terminus peptide: ERDKDNPNQYHYVA (ProteinTech Group, Inc) and anti-DDB1 was raised against a C-terminal fragment of recombinant DDB1 protein. Other antibodies used in this study are Cul1, Cul2 (Zymed), Elongin B, Skp1, HA (Santa Cruz), Flag (Sigma), Myc (Covance) and GST (Pharmacia). Anti-HA, anti-Flag and protein A sepharose beads were from Sigma.

### *In vitro *translation and GST pull down

One microgram of pHA-SAP130 DNA was in vitro transcribed/translated in the presence of S^35^-Methionine (Amersham) in 50 μl of TNT rabbit reticulocyte lysate system (RRL, Promega) according to the manufacturer's instructions. For experiments shown in Figures 2B, 2C and 6A, 25 μl of RRL containing HA-SAP130 or empty vector were incubated with 1 μg GST, GST-Ub or GST-Nedd8, or 1 μg GST-Nedd8 plus indicated amounts of GST or GST-CSN1 at room temperature for 30 min. The samples were then mixed with 75 μl of dilution buffer (50 mM Tris-HCl pH 7.5, 150 mM NaCl, 1.5 mM MgCl_2_, 0.1% NP-40, 1× protease inhibitor cocktail) and incubated with HA beads overnight at 4°C. The beads were washed five times with the dilution buffer and the samples were analyzed by western blot and autoradiography.

### *In vitro *ubiquitination assay

Samples were incubated at 30°C for 90 min in a 15 μl reaction containing 50 ng rabbit E1 (Boston Biochem), 100 ng GST-UbcH5b (Boston Biochem), 3 μg ubiquitin containing 1:2 ratio of biotinylated ubiquitin (Boston Biochem) or GST-Ub to unlabeled ubiquitin (Sigma), 50 mM Tris-HCl (pH 7.5), 5 mM MgCl_2_, 1 mM DTT and 4 mM ATP. For the in vitro ubiquitination assay shown in Figure 6B, each sample containing 2.5 mg of whole cell extract of a Flag-SAP130 line was incubated with indicated amounts of recombinant GST-CSN1, GST or purified porcine spleen CSN complex at room temperature for 30 min. Subsequently, Flag-SAP130 immunocomplex was isolated on beads. The beads were washed with lysis buffer prior to the *in vitro *ubiquitination assay as described above.

### Cell free de-neddylation inhibition assay

This procedure has been described by Yang *et al. *[24]. Briefly, total HeLa extract (80 μg) transfected with or without HA-Cul1 was supplied with ATP and ATP regeneration system. Anti-CSN2 antiserum (2 μl) or preimmune serum (2 μl) was added and incubated at room temperature for 15 min. The reactions were terminated by adding SDS loading buffer. The samples were blotted using anti-Cul1 and anti-HA antibodies.

## Abbreviations

CRL: cullin-RING ubiquitin ligase

CSN: COP9 Signalosome

SCF: Skp1-Cullin1-F-Box protein

RRL: Rabbit reticulocyte lysate

NTD: amino (N)-terminal domain

CTD: carboxyl (C)-terminal domain

HAT: histone acetyltransferase.

## Authors' contributions

The identification of CSN1 binding proteins was performed by TT. ND and KT performed mass-spec protein identification. The molecular and biochemical experiments on SAP130 were performed by SM with help from NW on plasmid constructions. Both SM and NW conceived and designed the experiments, and contributed to the writing of the manuscript. All authors read and approved the manuscript.
